# 517 Outcomes of Patients with Burns Associated with Home Oxygen Therapy: An Institutional Retrospective Review

**DOI:** 10.1093/jbcr/irac012.148

**Published:** 2022-03-23

**Authors:** Joshua S Yoon, Kimberly H Khoo, Joseph S Puthumana, Patrick R Keller, Tomer Lagziel, Carrie A Cox, Emily H Werthman, Panagis Galiatsatos, Charles S Hultman

**Affiliations:** R. Adams Cowley Shock Trauma Center, Division of Plastic Reconstructive and Maxillofacial Surgery, Herndon, Virginia; Department of Plastic and Reconstructive Surgery, Johns Hopkins University School of Medicine, Baltimore, Maryland; Johns Hopkins University Department of Plastic and Reconstructive Surgery, Baltimore, Maryland; Johns Hopkins University, Baltimore, Maryland; Department of Plastic and Reconstructive Surgery, Johns Hopkins School of Medicine, Baltimore, Maryland; Johns Hopkins University School of Medicine / Sackler School of Medicine, New-York Program, Tel-Aviv University, Rockville, Maryland; Johns Hopkins Bayview Medical Center, Baltimore, Maryland; Johns Hopkins Bayview Medical Center, Lutherville, Maryland; Division of Pulmonary and Critical Care Medicine, Johns Hopkins University, Baltimore, Maryland; Johns Hopkins University School of Medicine, Baltimore, Maryland

## Abstract

**Introduction:**

Home oxygen therapy (HOT) is frequently prescribed for patients with pulmonary dysfunction, which predisposes them to a unique health hazard at home. Prior studies show that HOT burns carry high morbidity and mortality, in large part due to inhalational injury. A significant portion of HOT patients are active smokers, which is the most frequent cause of HOT ignition. We conducted a retrospective review of patients with HOT related burns at our institution to characterize demographics and outcomes in this patient population.

**Methods:**

An IRB-approved single-institution retrospective review was conducted by querying our institutional burn registry for patients diagnosed with head and neck burns between July 2016 and January 2021. Patients with burns due to HOT ignition were included. Patients were separated into three groups: i) discharged from the emergency department (ED), ii) observed for less than 24 hours, and iii) admitted to the hospital. Demographic and clinical outcome data were compared between groups.

**Results:**

We identified 100 patients with HOT burns, who were evaluated from 2016-2021, during which time we treated 3606 patients with burn injuries. Mean age was 66.6 ± 9.3 years with a male to female ratio of 1.3:1 and median TBSA of 1%. In these patients, 97% were on HOT for COPD and smoking caused 87.3% of burns. Thirteen were discharged from the ED, 35 observed for less than 24 hours, and 52 admitted. For admitted patients, 69.2% were admitted to the ICU with a median ICU stay of 1.5 days, 37% required intubation for a median duration of 1 day, and 11.5% required debridement and grafting with an average of 2.6 ± 1.6 procedures. Inhalational injury was found in 26.9% of patients, 3.9% underwent tracheostomy, and 17.3% experienced hospital complications. In-hospital mortality was 9.6% and 7.7% discharged to hospice. Among those admitted, median length of stay was 4 days and 67.3% discharged home. After discharge, 13.5% required readmission within 30 days. Patients admitted to the hospital had significantly higher rates of admission to the ICU, intubation, and inhalational injury compared to those that were not admitted (p < .01).

**Conclusions:**

Most HOT-related burns are caused by smoking and these injuries can result in significant morbidity and mortality. Efforts to educate and encourage smoking cessation with more judicious HOT allocation would assist in preventing these unnecessary highly morbid injuries.

